# Sex-Based Classification of Grey Matter Volume Using SVM: Implications for Early-Phase Psychotic and Mood Disorders

**DOI:** 10.1192/j.eurpsy.2025.1672

**Published:** 2025-08-26

**Authors:** M. F. Urquijo, A. Riecher-Rössler, P. Falkai, D. Dwyer, P. Consortium, N. Koutsouleris

**Affiliations:** 1 Psychiatry Dept., University Hospital, LMU Munich, Munich, Germany; 2 Medical Faculty, University of Basel, Basel, Switzerland; 3 Orygen, University of Melbourne, Melbourne, Australia; 4 Psychiatry Department, University hospital Cologne, Cologne; 5 University of Münster, Münster, Germany; 6 Aldo Moro University of Bari, Bari; 7 University of Udine, Udine, Italy; 8 University of Turku, Turku, Finland; 9 University of Düsseldorf, Düsseldorf, Germany; 10 University of Melbourne, Melbourne, Australia; 11 Psychiatry Dept., King’s College, London, United Kingdom; 12 Max Planck Institute for Psychiatry, Munich, Germany

## Abstract

**Introduction:**

Understanding sex differences in brain structure is crucial for advancing personalized medicine. However, the alterations of sex-based neuroanatomical differences during the early phases of mood and psychotic disorders remain largely underexplored.

**Objectives:**

This study aimed to evaluate neuroanatomical sex-based differences in a healthy cohort and assess potential alterations in clinical populations from the early psychiatric spectrum using a machine learning approach.

**Methods:**

For this, we developed a Support Vector Machine (SVM) model trained to classify sex based on grey matter volume from a cohort of healthy controls (HC, n = 521), aged 15 to 40 years. After optimization and cross-validation, the model was applied to three distinct clinical populations from the same age range: individuals at clinical high risk for psychosis (CHR, n =334), those with recent onset psychosis (ROP, n = 351), and those with recent onset depression (ROD, n = 309).

**Results:**

The model achieved a robust balanced accuracy (BAC) of 85.1% (p < .01) in the HC cohort. When applied to the CHR group, the model maintained a BAC of 85.5%, whereas its performance decreased in the ROP (75.8% BAC) and ROD (79.3% BAC) groups. In contrast to previous literature, female participants in the ROP group exhibited a masculinization of brain patterns, while male participants showed no such reversal. In the ROD group, both sexes revealed a slight tendency toward patterns typical of the opposite sex.

**Conclusions:**

These preliminary findings suggest that sex-based neuroanatomical structures remain preserved at high-risk stages but undergo alterations with disorder progression. Future research will investigate the observed performance decline in relation to other phenotypic factors. These findings offer novel insights into sex-specific neurobiological mechanisms underlying psychotic and mood disorders and their early markers.

**Disclosure of Interest:**

None Declared

